# Genome Sequence of Salmonella enterica Serovar Typhimurium Bacteriophage MG40

**DOI:** 10.1128/MRA.00905-20

**Published:** 2020-09-10

**Authors:** Eddie B. Gilcrease, Justin C. Leavitt, Sherwood R. Casjens

**Affiliations:** a Division of Microbiology and Immunology, Department of Pathology, University of Utah School of Medicine, University of Utah, Salt Lake City, Utah, USA; b School of Biological Sciences, University of Utah, Salt Lake City, Utah, USA; DOE Joint Genome Institute

## Abstract

We report the complete genome sequence of P22-like Salmonella enterica serovar Typhimurium phage MG40, whose prophage repressor specificity is different from that of other known temperate phages.

## ANNOUNCEMENT

MG40 was isolated from human stool in the mid-1960s and is a short-tailed double-stranded DNA generalized transducing phage (1). Wild-type MG40 was obtained from David Botstein and propagated on Salmonella enterica DB7000 (2) grown in LB broth (3) at 37°C. MG40 DNA was purified from CsCl gradient-purified virions by the method described by Casjens and Gilcrease (4). An Illumina TruSeq library was prepared using a TruSeq DNA PCR-free HT library preparation kit and sequenced using Illumina MiSeq 150-bp paired-end run methodology with a 350-bp insert library. The reads obtained were quality controlled using FastQC (www.bioinformatics.babraham.ac.uk/projects/fastqc). Geneious v9.0.5 was used for trimming and assembly of the reads; not including trimming and including trimming both gave identical sequences, and the Geneious *de novo* assembly program was used to assemble the sequence (5). A single circular contig with 373.3× mean coverage was obtained. Circular sequence assembly is expected for headful packaging phages (6, 7). The genome was annotated using the annotation pipeline hosted by the Center for Phage Technology (https://cpt.tamu.edu/galaxy-pub). All tools are hosted in the Galaxy and Web Apollo platforms and, unless otherwise stated, were executed using default parameters (8, 9). No tRNAs were detected using ARAGORN v2.36 (10). Rho-independent transcription termination sites were annotated using TransTermHP v2.09 (11). GLIMMER v3.0 and MetaGeneAnnotator v1.0 were used to predict protein-coding genes (12, 13). The prediction of gene functions was facilitated by InterProScan v5.33-72, TMHMM v2.0, LipoP v1.0, and BLAST v2.2.31 searches against the NCBI nonredundant, UniProtKB, Swiss-Prot, and TrEMBL databases (14–18).

The MG40 genome is 40,315 bp long (47.2% G+C content), and we annotated 72 genes in its chromosome. MG40 is relatively unstudied, but its virions are essentially indistinguishable from those of P22 by negative-stain electron microscopy (1), and we found that its virion assembly genes are indeed similar to those of P22 (GenBank accession no. BK000583); however, many of its other genes are quite different, and its genome is mosaically related to that of P22. MG40 was shown to have different repressor specificity and the same host chromosome integration site as P22 (1), and its repressor gene is very different from those of the studied P22-like phages, while its integrase is nearly identical to that of P22. MG40 has only 156 bp in place of the ∼3-kbp P22 *immI* region, and its genome encodes a putative prophage-expressed O-antigen rhamnoseacetylase that is 92% identical to the characterized homologue of P22-like phage BTP1 (19); the P22 genome encodes three proteins (GtrA, GtrB, and GtrC) that add glucose to the O-antigen at this genome location. Curiously, although it infects S. enterica serovar Typhimurium and, like P22, requires O-antigen for infection (1), the C-terminal 464-amino-acid region of the receptor-binding domain of its tailspike is only 33% identical to that region of the phage P22 tailspike, while it is 99.6% identical to the putative phage SPN9TCW tailspike (GenBank accession no. JQ691610). Both phages infect S. enterica serovar Typhimurium, but SPN9TCW is a member of the ε15-like cluster of phages (20), which have a number of differences from the P22-like phages.

### Data availability.

The genome sequence and associated data for the phage MG40 genome are available in GenBank under accession no. MT774487, BioProject no. PRJNA646767, SRA accession no. SRR12282813, and BioSample no. SAMN15565527.
